# Fission Yeast Apc15 Stabilizes MCC-Cdc20-APC/C Complexes, Ensuring Efficient Cdc20 Ubiquitination and Checkpoint Arrest

**DOI:** 10.1016/j.cub.2017.03.013

**Published:** 2017-04-24

**Authors:** Karen M. May, Flora Paldi, Kevin G. Hardwick

**Affiliations:** 1 Wellcome Trust Centre for Cell Biology, University of Edinburgh, King’s Buildings, Max Born Crescent, Edinburgh EH9 3BF, UK

**Keywords:** mitosis, spindle checkpoint, APC/C, MCC, Cdc20, Apc15, Apc14, Mad3, mitotic checkpoint, spindle assembly checkpoint

## Abstract

During mitosis, cells must segregate the replicated copies of their genome to their daughter cells with extremely high fidelity. Segregation errors lead to an abnormal chromosome number (aneuploidy), which typically results in disease or cell death [1]. Chromosome segregation and anaphase onset are initiated through the action of the multi-subunit E3 ubiquitin ligase known as the anaphase-promoting complex or cyclosome (APC/C [2]). The APC/C is inhibited by the spindle checkpoint in the presence of kinetochore attachment defects [3, 4]. Here we demonstrate that two non-essential APC/C subunits (Apc14 and Apc15) regulate association of spindle checkpoint proteins, in the form of the mitotic checkpoint complex (MCC), with the APC/C. *apc14Δ* mutants display increased MCC association with the APC/C and are unable to silence the checkpoint efficiently. Conversely, *apc15Δ* mutants display reduced association between the MCC and APC/C, are defective in poly-ubiquitination of Cdc20, and are checkpoint defective. In vitro reconstitution studies have shown that human MCC-APC/C can contain two molecules of Cdc20 [5, 6, 7]. Using a yeast strain expressing two Cdc20 genes with different epitope tags, we show by co-immunoprecipitation that this is true in vivo. MCC binding to the second molecule of Cdc20 is mediated via the C-terminal KEN box in Mad3. Somewhat surprisingly, complexes containing both molecules of Cdc20 accumulate in *apc15Δ* cells, and the implications of this observation are discussed.

## Highlights

• Two small fission yeast APC/C subunits regulate MCC binding
• Apc14 is required for checkpoint silencing and Apc15 for checkpoint arrest
• Apc15 enhances Cdc20 ubiquitination and turnover
• Two Cdc20 molecules are found in MCC-APC/C and free MCC-Cdc20^A^ accumulates in *apc15Δ*

## Graphical Abstract

**Figure:**
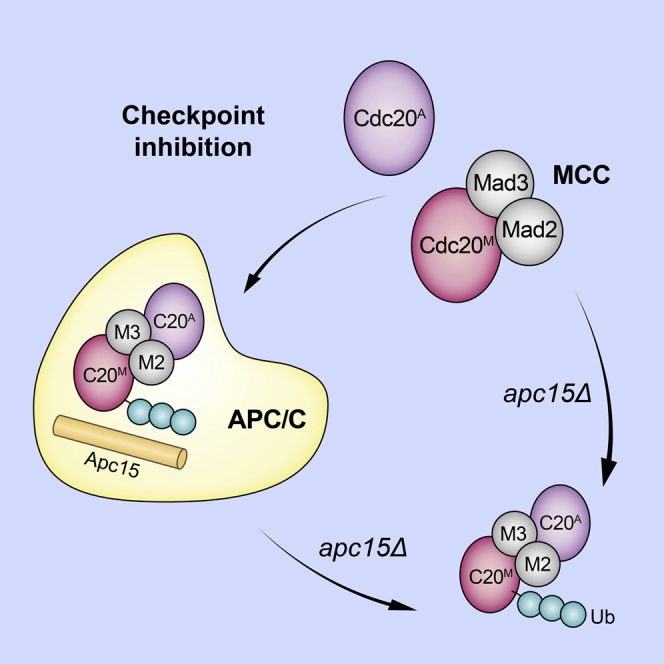

## Teaser

May et al. dissect the roles of fission yeast Apc14 and Apc15 in spindle checkpoint regulation of APC/C activity. Apc15 is needed for efficient Cdc20 turnover and checkpoint arrest in fission yeast. Apc15 and the C terminus of Mad3 are shown to co-ordinate the interactions of two molecules of Cdc20 within inhibited APC/C complexes.

## Results and Discussion

Cdc20^Slp1^ (Slp1 is the fission yeast homolog of Cdc20) was identified several years ago as the key effector of the spindle checkpoint [8, 9], and the mitotic checkpoint complex (Cdc20-Mad3-Mad2-Bub3) was found to be the most potent anaphase-promoting complex or cyclosome (APC/C) inhibitor [10]. The crystal structure of fission yeast mitotic checkpoint complex (MCC) has been solved [11], and recently cryoelectron microscopy (cryo-EM) structures of human MCC in complex with APC/C were obtained from recombinant complexes produced in baculovirus-infected insect cells [6, 7]. These models provide an excellent structural framework within which spindle checkpoint inhibition takes place, yet there are several areas requiring further insight. The Gould laboratory previously demonstrated that deletion of either the Apc14 or Apc15 subunit of fission yeast APC/C did not affect its ubiquitin ligase activity [12]. Apc14 is not well conserved, but in human and budding yeast cells it has been shown that there are spindle checkpoint silencing defects in the absence of Apc15 [13, 14, 15]. Therefore, we analyzed the fission yeast *apc14Δ* and *apc15Δ* strains for defects in checkpoint establishment, maintenance, and/or silencing.

### *apc15*Δ Mutants Display Spindle Checkpoint Defects

**Figure 1:**
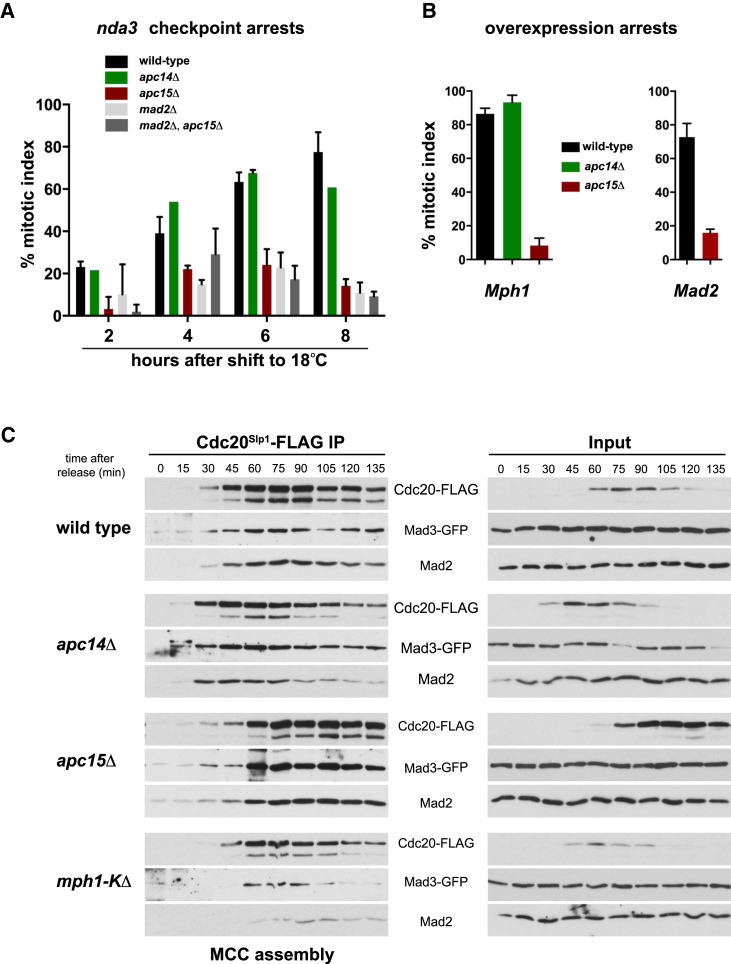
*apc15Δ* Mutants Are Checkpoint Defective (A) Checkpoint arrest. *nda3* strains were grown to log phase and then shifted to 18°C to de-polymerize microtubules and thereby activate the spindle checkpoint. At the time points indicated, cells were fixed in methanol and the mitotic index was scored by analyzing the levels and localization of Cdc13-GFP (cyclin B). Cdc13-GFP localizes to the spindle pole bodies in early mitosis. The *apc14Δ* mutant arrests proficiently, but *apc15Δ* and *mad2Δ* mutants do not. This experiment was repeated three times (with at least 100 cells scored per strain at each time point), and the data are plotted as the mean ± SD. (B) Mad2 and Mph1 overexpression. Cultures containing plasmids expressing Mad2 from the *nmt1* promoter or Mph1 from the *nmt41* promoter were induced (−thiamine) for 18 hr and the mitotic index was scored by immunostaining of microtubules and spindle length. This experiment was repeated twice (with at least 100 cells scored per strain at each time point), and the data are plotted as the mean ± SD. (C) MCC assembly is not affected. *cdc25-22 cdc20-FLAG* cultures were synchronized at G2/M by cdc25 block and release, cell samples were taken at 15-min intervals, and Cdc20 was subjected to immunoprecipitation (IP) and analyzed for associated checkpoint proteins (Mad3 and Mad2). This experiment was repeated three times, and a representative example is shown here. See also Figure S1.

First, we employed the cold-sensitive beta-tubulin mutant *nda3-KM311* to analyze the ability of cells to arrest in mitosis in the absence of spindle microtubules [16]. Figure 1A demonstrates that whereas *apc14Δ* cells arrest like wild-type, *apc15Δ* cells display severe defects in this checkpoint assay. We also analyzed the ability of these *apc* mutants to arrest in response to Mad2 and Mps1^Mph1^ (Mph1 is the fission yeast homolog of Mps1 kinase) overexpression [17, 18], and once again found that *apc15Δ* strains were significantly defective in arrest (Figure 1B). The inability to respond to Mad2 overexpression places Apc15 function downstream in the spindle checkpoint pathway, as the only other checkpoint protein required for the Mad2 overexpression arrest is Mad3, the other key component of fission yeast MCC [19].

This suggested that Apc15 might have a role in MCC assembly and/or its association with the APC/C. To test this, we analyzed MCC assembly in an unperturbed mitosis, having synchronized cells at G2/M using the *cdc25-ts* allele. Figure 1C shows that there is little effect on MCC assembly in the absence of either Apc14 or Apc15. However, we note that in the *apc15Δ* mutant, the levels of Cdc20^Slp1^ and the MCC complex are 2- to 3-fold higher and stay high for longer, even though they display no delay in anaphase (see Figure S1).

**Figure 2:**
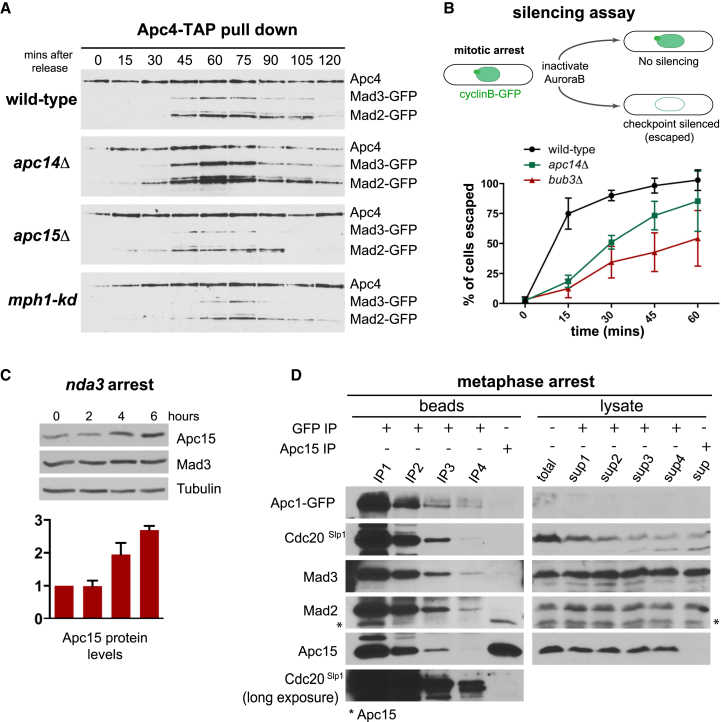
*apc14Δ* and *apc15Δ* Mutants Both Perturb the Interaction between MCC Complexes and the APC/C, but in Opposite Ways (A) APC/C binding time courses. *cdc25-22 apc4-TAP mad3-GFP* cultures were synchronized at G2/M by *cdc25* block and release, cell samples were taken at 15-min intervals, and Apc4-TAP was pulled down and analyzed for associated checkpoint proteins (Mad3 and Mad2). The cells from each time point were fixed in methanol and the number of binucleate cells was determined by DAPI staining DNA. This experiment was repeated twice, and a representative example is shown here. (B) Checkpoint silencing assay. *nda3KM-311 cdc13-GFP* strains were arrested by shifting to 18°C to de-polymerize microtubules and thereby activate the spindle checkpoint. The Ark1-as kinase was then inhibited with 5 μM 1NM-PP1 and live-cell samples were analyzed at 15-min intervals. Ark1 inhibition activates the APC, and Cdc13-GFP is rapidly degraded in wild-type cells. The mitotic index was scored in live cells by analyzing the levels and localization of Cdc13-GFP (cyclin B). In arrested cells, this is nuclear with a bright signal at the spindle pole bodies (SPBs). The number of cells that degrade Cdc13-GFP is shown as a percentage of arrested cells at t = 0. This experiment was repeated at least three times (with at least 100 cells scored per strain at each time point), and the data are plotted as the mean ± SD. (C) *nda3-KM311* mutants were grown to log phase and then shifted to 18°C for 6 hr, taking time points at 2-hr intervals. Whole-cell immunoblots were then analyzed for levels of Apc15 Mad3 and tubulin. This experiment was repeated three times, and the data are plotted as the mean ± SD. See Figure S2B for quantitation of Apc15 levels through the cell cycle. (D) Cells were arrested in metaphase, through *nda3* arrest, and lysates were prepared and then immunodepleted for APC/C complexes through four rounds of Apc1-GFP immunoprecipitation. Apc15 was then immunoprecipitated from the final supernatant. The five sets of beads were then analyzed for associated Cdc20 and checkpoint proteins, revealing high levels of free Apc15 after APC/C depletion. This experiment was repeated twice. See also Figure S2.

When we analyzed the ability of the MCC complex to bind to the APC/C, we saw striking effects in both the *apc14Δ* and *apc15Δ* strains (Figure 2A). Cells were synchronized at G2/M and then released into mitosis. The levels of MCC bound to the APC/C in *apc15Δ* strains were reduced to a level similar to those in the absence of Mps1^Mph1^ kinase activity [20]. Conversely, the MCC levels bound in *apc14Δ* strains were 2- to 3-fold higher than in wild-type cells (see Figure S2 for quantitation). The simplest interpretation of these data is that Apc15 is required for stable MCC binding to the APC/C, which is consistent with the checkpoint defects observed in *apc15Δ* strains (Figure 1), whereas Apc14 function is required for efficient MCC release and checkpoint silencing.

### *apc14Δ* Mutants Display Checkpoint Silencing Defects

To analyze the *apc14Δ* phenotype in more detail, we employed a checkpoint silencing assay that we previously developed during studies of protein phosphatase 1 [21]. Cells are arrested without microtubules using the *nda3* mutation [16], and then Aurora activity is inhibited using the *ark1-as3* allele [22]. Because Ark1 activity is necessary to maintain spindle checkpoint arrests [21, 23], these cells rapidly degrade cyclin B. Figure 2B shows that there is a clear delay in cyclin B degradation in *apc14Δ* cells, consistent with the hypothesis that Apc14 has a role in checkpoint silencing. This Apc14 silencing function is the subject of ongoing work, but was not analyzed further in this study.

### Apc15 Is Cell-Cycle Regulated

Transcript data in PomBase [24] show that Apc15 expression is tightly cell-cycle regulated, with highest expression at G2/M. Therefore, we analyzed the cell-cycle levels of the Apc15 protein. Apc15 is a rather small protein, and we were concerned that epitope tags might perturb its function. Therefore, we generated specific, polyclonal antibodies to Apc15. Figure S2B shows that the levels of Apc15 increase ∼2-fold during mitosis, and Figure 2C demonstrates that Apc15 levels increase further during prolonged checkpoint arrest. As is the case for many APC/C subunits, Apc15-GFP accumulates in the nuclei of mitotic cells [25]. We carried out several experiments to test whether the Apc15 newly synthesized at G2/M is important for checkpoint function and whether Apc15 is itself subject to ubiquitin-mediated degradation. As yet, we have found no clear evidence that this regulation is physiologically important for mitosis (see Figure S2).

At this point, we had two simple hypotheses for Apc15 function: (1) Apc15p could act as a loading factor, by first binding to the MCC and then helping it associate with the APC/C core particle, or (2) Apc15 might form an important part of the MCC docking site on the APC/C. To distinguish between these modes of action, we analyzed the association of Apc15 with the APC/C and with MCC proteins in yeast lysates. To test whether there was a free pool of Apc15p and whether this could interact with the MCC, we carried out immunodepletion of Apc1-GFP from mitotic extracts. As expected, Apc15 co-immunoprecipitates with Apc1-GFP (Figure 2D). No Apc15 remains associated with Apc1-GFP in an extract after three rounds of Apc1-GFP immunodepletion, yet there is still a significant pool of free Apc15. Importantly, when we immunoprecipitated this free pool of Apc15 it was not associated with Mad3, Mad2, or Cdc20 (Figure 2D). This experiment indicates that although there was a significant non-APC/C-bound pool of Apc15, it was not associated with the MCC complex or proteins therein. This argues against the model where Apc15 acts as an MCC loading factor, as it would be expected to bind MCC components independent of APC/C binding. The role of the free Apc15 pool remains unclear, but its existence most likely explains why there was no significant phenotype upon transcriptional depletion of Apc15 (Figures S2D–S2F).

### Fission Yeast Apc15 Is Required for Efficient Cdc20 Ubiquitination

**Figure 3:**
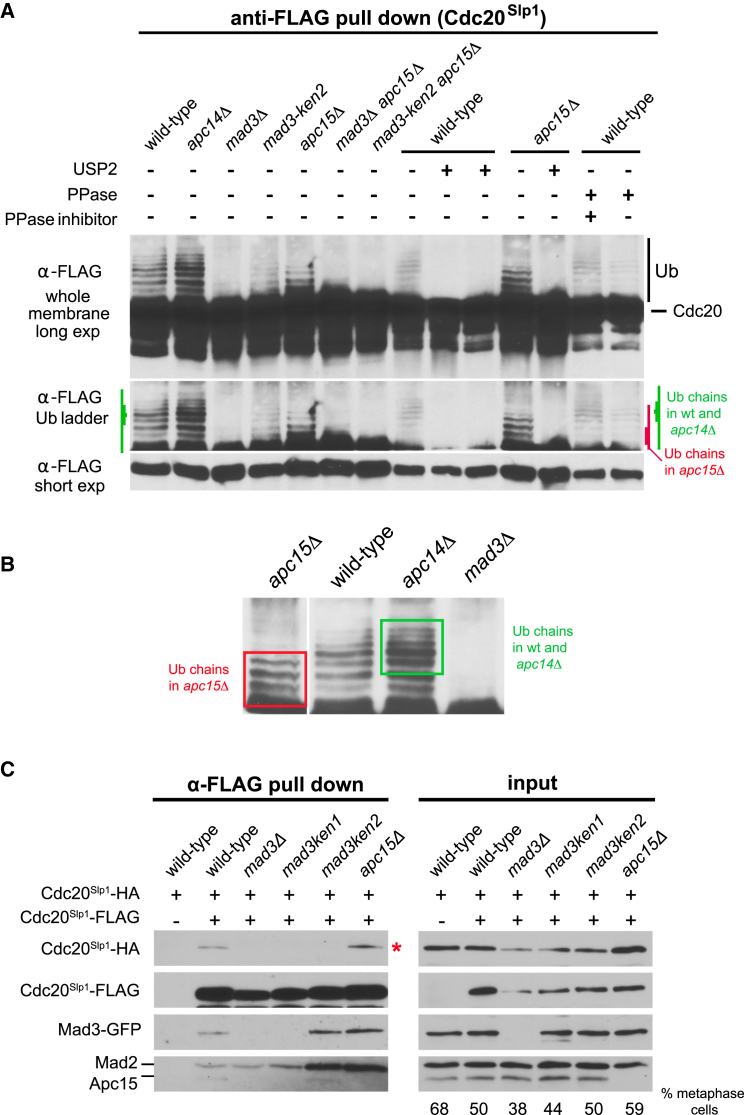
*apc15Δ* Mutants Have Significant Defects in the Processivity of Cdc20^Slp1^ Ubiquitination, and Fission Yeast MCC-Cdc20-APC/C Contains Two Molecules of Cdc20 (A) Cdc20 ubiquitination experiments. The indicated strains all contain the *mts3-1* proteasome mutation, to block cells in mitosis independent of the spindle checkpoint and to enrich for poly-ubiquitinated forms of cellular proteins. Cultures were shifted to 36°C 3 hr prior to harvesting. Whole-cell lysates were made in the presence of Dub inhibitors, and Cdc20-FLAG was immunoprecipitated and then immunoblotted for Cdc20-FLAG. Long exposure reveals a ladder of slow-migrating bands, which are reduced in *mad3* and *apc15* mutants. The indicated lysates were treated with recombinant hsUSP2 (de-ubiquitinase) or lambda phosphatase prior to running the gel. Lambda phosphatase has no effect but USP2 abolishes the ladder, confirming that this is due to modification with ubiquitin. Different modified forms of Cdc20 accumulate in the *mts3* and *apc15Δmts3* mutants, with shorter chains in the absence of Apc15. These are indicated with green and red markings by the relevant anti-ubiquitin blots. This experiment was repeated three times. (B) The indicated lanes from (A) are expanded to highlight the different modified forms of Cdc20 that accumulate in the *mts3*, *apc15Δmts3*, and *apc14Δmts3* mutants. Most notably, there are shorter chains in the absence of Apc15 (boxed in red). (C) Cdc20-FLAG and Cdc20-HA co-immunoprecipitate. Cells containing both Cdc20 forms were synchronized in mitosis (60 min after *cdc25* block and release), lysates were prepared, and Cdc20-FLAG was immunoprecipitated. The immunoprecipitates were then immunoblotted and analyzed for associated Cdc20-HA, Mad3-GFP, Mad2, and Apc15. The asterisk indicates Cdc20-Cdc20 co-immunoprecipitation. This experiment was repeated three times. The % of metaphase cells is indicated below the blot for the six strains. See also Figure S3.

Deletion of Apc15 had no effect on the ability of cells to assemble the MCC, but we noted that the levels of Cdc20 and MCC remained high for longer in *apc15Δ* than in wild-type cells (Figure 1C). This suggested that Apc15 might regulate Cdc20 protein levels and that it could be required for efficient Cdc20 ubiquitination and/or degradation. To determine whether this was the case, we arrested cells in metaphase using *mts3-1* [26], a proteasome mutant that does not require a functional checkpoint for arrest, and then immunoprecipitated Cdc20-FLAG. It was hoped that the *mts3-1* mutation would stabilize ubiquitin-modified forms of Cdc20, due to defective proteasome action. When we probed immunoblots for Cdc20, we could now detect a discrete ladder of slower-migrating bands (Figures 3A and 3B). Addition of phosphatase had no effect on these bands, but the ladder was removed in extracts treated with the recombinant de-ubiquitinase USP2 [27]. This demonstrates that Cdc20 accumulates in poly-ubiquitinated forms in these mitotically arrested *mts3* cells. Poly-ubiquitination of Cdc20 is dependent on MCC formation [28, 29]. As expected, in *mad3Δ* and *mad2Δ* strains, which are unable to form the MCC, Cdc20 ubiquitination was largely abolished. Importantly, in *apc15Δ* cells, Cdc20 was still ubiquitinated, but there was an accumulation of Cdc20 molecules with shorter (approximately one to four) ubiquitin (Ub) chains (Figure 3B). This suggests that in fission yeast, deleting Apc15 does not block the initial binding of the MCC to the APC/C and the subsequent ubiquitination of Cdc20 but that Apc15 is required for *processive* ubiquitination of Cdc20. This is consistent with our observation that less MCC binds to APC/C in *apc15Δ* cells (Figure 2A). We propose that the MCC frequently “falls off” an APC/C particle lacking Apc15 before longer chains of ubiquitin can be added to Cdc20, leading to reduced processivity of Cdc20 ubiquitination. As a consequence, the Cdc20 protein is stabilized in *apc15Δ* cells and free MCC levels accumulate (see Figures S1A and S1B).

Further analysis showed that *mad3-ken2* mutants also displayed reduced ubiquitination, and that the *mad3-ken2 apc15Δ* double mutant is completely unable to poly-ubiquitinate Cdc20 (Figure 3A). This suggests that Mad3-KEN2 and Apc15 are both involved in stabilizing key MCC-APC/C interactions in fission yeast that are required for efficient Cdc20 ubiquitination and for checkpoint arrest. This agrees well with the accompanying manuscript, where KEN2 and its conserved flanking ABBA motifs are all shown to enhance Cdc20-APC/C inhibition and spindle checkpoint arrest [30].

### Fission Yeast MCC Can Contain Two Cdc20 Molecules, and Binding of the Second Molecule Is Mad3-KEN2 but Not Apc15 Dependent

Musacchio and co-workers proposed a few years ago that a second molecule of Cdc20 in MCC complexes would help explain the need for two conserved KEN boxes in Mad3/BubR1 and aspects of Cdc20 turnover [2, 29]. It was then argued from in vitro studies that human MCC can bind a second molecule of Cdc20, enabling it to inhibit active Cdc20-APC/C [5]. Recent cryo-EM studies have demonstrated that when human MCC-APC/C is reconstituted in insect cells there are indeed MCC complexes that contain two Cdc20 molecules [6, 7]. To distinguish the two Cdc20 molecules, we will refer to them as the MCC-bound form (Cdc20^M^) and the APC/C-bound or activator form (Cdc20^A^).

To provide formal proof of the second Cdc20 molecule in a living system, we generated a fission yeast strain containing a second copy of the Cdc20^Slp1^ gene. This copy has an internal 3×FLAG tag [31], enabling it to be readily distinguished from the first copy, which has a C-terminal 3×HA (hemagglutinin) tag. Both of these forms of Cdc20^Slp1^ are functional and bind the APC/C (see below). If two copies of Cdc20 are present in the MCC, then the two proteins would be co-immunoprecipitated from mitotic extracts. Figure 3B shows that this is the case, that their interaction is dependent on Mad3p, and importantly that their interaction is dependent on both of the conserved Mad3-KEN boxes. The first KEN box (KEN1) is known to be critical for direct Cdc20 binding and MCC formation [11], but the function of the second KEN box (KEN2) is less well understood [32]. KEN2 is widely recognized as being necessary for checkpoint function but not for MCC formation. In BubR1, it can compete with substrates for APC/C interaction in vitro [32]. Our finding that Mad3-KEN2 is needed for interaction with the second molecule of Cdc20 is in agreement with the human study where the D box and the second KEN box in BubR1 were both found to be necessary [5] and with the recent cryo-EM structures [6, 7]. Importantly, our Cdc20-Cdc20 co-immunoprecipitation experiment is carried out in whole-cell extracts made from mitotic fission yeast cells. Our findings are confirmed in the accompanying fission yeast manuscript [30], where two forms of Cdc20 (one tagged and one untagged) are shown to interact in mitosis. One way to form these Cdc20-Cdc20 complexes would be for the MCC as a whole to form dimers, either simply with itself or on a larger platform. In this model, all members of the MCC complex would contain two molecules, rather than just Cdc20. To test this, we engineered fission yeast strains expressing normal Mad3 and Mad3-GFP, and asked whether the two forms of Mad3 can be co-immunoprecipitated in mitotically arrested cells. Figure S3B shows that this was not the case, ruling out MCC dimerization.

**Figure 4:**
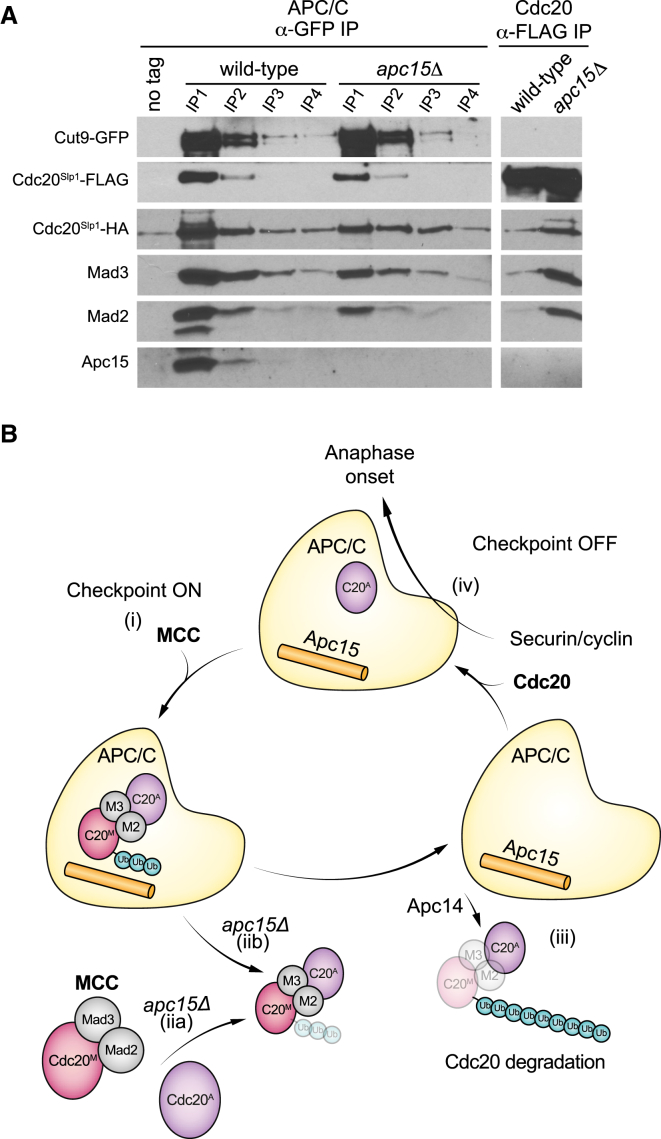
A Free Pool of Cdc20^M^-Mad3-Mad2-Cdc20^A^ Accumulates in *apc15Δ* Mutants (A) The MCC (Cdc20^M^-Mad3-Mad2)-Cdc20^A^ complex can be found in the APC/C-depleted supernatant, and the complex accumulates in *apc15Δ*. Mitotic lysates were prepared from *cdc25-22 cdc20-HA cdc20-FLAG Apc6*^*Cut9*^*-GFP* (60 min after *cdc25* block and release) and then immunodepleted for APC/C complexes through four rounds of *cut9*^*apc6*^-GFP immunodepletion. Cdc20-FLAG was then immunoprecipitated from the resulting supernatant and immunoblotted to look for associated Cdc20-HA and checkpoint proteins. The MCC (Cdc20^M^-Mad3-Mad2)-Cdc20^A^ is immunoprecipitated without Apc6^Cut9^-GFP or Apc15. This experiment was repeated twice. (B) Models of MCC binding and Cdc20 ubiquitination, in wild-type cells and *apc15* mutants. (i) When the checkpoint is on, the MCC binds to Cdc20-APC/C and in fission yeast this interaction is stabilized by Apc15. The C terminus of Mad3 (KEN2 and associated ABBA motifs) is critical for this stable interaction with the second molecule of Cdc20 (Cdc20^A^). (iia) In *apc15Δ* cells, the MCC could preferentially bind free Cdc20^A^. (iib) In the absence of Apc15, the MCC complex is weakly bound and Cdc20^M^ is inefficiently ubiquitinated. It is released with short ubiquitin chains in the form of Cdc20^M^-Mad3-Mad2-Cdc20^A^. Note that both molecules of Cdc20 are released from the APC/C. (iii) In wild-type cells Cdc20^M^ is efficiently poly-ubiquitinated, leading to its degradation. Apc14 function is required for efficient release of the MCC. (iv) The checkpoint is off; APC/C is now free to be bound by the Cdc20^A^ activator, which can catalyze the poly-ubiquitination of securin and cyclin, leading to anaphase onset. See also Figure S4.

The model proposed in the human studies [5] was that the MCC (Cdc20^M^-Mad3-Mad2) binds and inhibits an active Cdc20^A^-APC/C complex to form Cdc20^M^-Mad3-Mad2-Cdc20^A^-APC/C (containing two Cdc20 molecules). Our *apc15* mutant allowed us to test this model in fission yeast. If this were the case, one would predict that the *apc15* mutant, which impairs the interaction between the MCC and APC/C, will reduce or abolish the interaction between the two Cdc20 molecules. Figure 3B shows that this is not the case, as efficient co-immunoprecipitation of Cdc20-FLAG and Cdc20-HA was still observed in *apc15Δ* cells; indeed, these complexes accumulated in the mutant (see also Figure S3A). Figure 2A demonstrates that there was a significant reduction in the level of MCC bound to the APC/C in *apc15Δ* mutants, yet Figure 3B reveals complexes containing multiple Cdc20 molecules. To confirm that these Cdc20 complexes were not associated with the APC/C, we depleted the APC/C from *apc15Δ* extracts via four rounds of Cut9-GFP immunodepletion and asked whether the free pool of MCC still contained two molecules of Cdc20 (Cdc20-HA–Mad3–Mad2–Cdc20-FLAG). Figure 4A shows that this is the case, and that there is significantly more of this complex in *apc15Δ* mutant extracts compared to wild-type. We note that this observation is not necessarily in line with the proposed human model of Cdc20-APC/C inhibition [5], and suggest two models to explain our fission yeast observations:

(1) Apc15 forms an important part of the MCC binding site on the APC/C. As a consequence, in *apc15Δ* cells the MCC (Cdc20^M^-Mad3-Mad2) might preferentially bind to free Cdc20 rather than to Cdc20^A^-APC/C complexes. This will sequester Cdc20 and form a free pool of Cdc20^M^-Mad3-Mad2-Cdc20^A^. Such sequestration may well help inhibit Cdc20 action, and the accompanying manuscript [30] shows this to be the case when Cdc20 levels are reduced. However, we note that the Cdc20^M^ in these complexes would not be ubiquitinated unless the complex then bound to the APC/C.
(2) The MCC binds to Cdc20^A^-APC/C, but their interaction is weakened in the absence of Apc15 and the MCC-Cdc20^A^-APC/C complex is rather short-lived. A consequence of this brief interaction with the APC/C is that it would lead to a reduced processivity of Cdc20^M^ ubiquitination in *apc15Δ* cells. This is consistent with the short Ub chains we have observed on Cdc20 in *apc15Δ* (Figure 3B). Importantly, when the MCC complex dissociates it takes the Cdc20^A^ activator with it to form free Cdc20^M^-Mad3-Mad2-Cdc20^A^.

Note that these models are not mutually exclusive. It is important to include the free Cdc20^M^-Mad3-Mad2-Cdc20^A^ complex in overviews of MCC action and Cdc20 inhibition (see Figure 4B).

### Conclusions

At first glance, fission yeast Apc15 appears to have a simple role to play in helping the MCC stably bind to the APC/C core particle, and this is consistent with its position in recent high-resolution cryo-EM structures of the APC/C. Budding yeast and in vitro human studies have argued that Apc15 is needed for efficient Cdc20 ubiquitination and subsequent MCC release [13, 15]. Based on the cryo-EM models, it was proposed that human Apc15 undergoes a conformational change to shift the MCC into a suitable orientation for Cdc20 auto-ubiquitination [6, 7]. Our study is consistent with this, as Cdc20^Slp1^ displays reduced ubiquitination and is stabilized in fission yeast *apc15Δ* cells (Figure 3A). However, fission yeast Apc15 is also needed for stable MCC binding and thus for spindle checkpoint arrest, which is not the case in human and budding yeast cells.

Importantly, we also provide in vivo evidence for complexes containing two Cdc20 molecules in fission yeast. Interaction with the second molecule of Cdc20 is mediated by the C terminus of Mad3, including its second KEN box and the nearby ABBA motifs [32, 33, 34]. Our work and the accompanying manuscript [30] both describe in vivo fission yeast studies that provide an important confirmation of models proposed from in vitro reconstitution studies of human MCC proteins and their interactions with the APC/C. New findings are presented here where, paradoxically, although *apc15Δ* mutants display reduced levels of MCC bound to the APC/C (Figure 2A), they also accumulate Cdc20-Cdc20 complexes (Figure 3B). We have suggested two models for generating the free pool of Cdc20^M^-Mad3-Mad2-Cdc20^A^ that we observe in *apc15Δ* mutants (see Figure 4B).

In fission yeast, we find that deletion of Apc14 leads to checkpoint silencing defects. In its absence there are higher than normal levels of the MCC bound to the APC/C, and experiments are ongoing to understand its mode of action. It will be interesting to see whether in the absence of Apc14 the MCC simply binds more tightly to the APC/C, or whether Apc14 has an active role similar to p31^comet^ (not conserved in *S. pombe*), TRIP13, or the CCT chaperone in disrupting MCC-APC/C complexes [35, 36].

## Experimental Procedures

See the Supplemental Experimental Procedures.

## Author Contributions

K.M.M. conceived and designed the experiments, acquired data, performed analysis and interpretation of the data, and drafted the figures. F.P. generated Slp1 constructs and yeast strains and performed certain Cdc20 co-immunoprecipitations. K.G.H. conceived the project, helped with analysis and interpretation of the data and figure construction, and wrote the manuscript. All authors reviewed the manuscript.

## Supplemental Information

Document S1. Supplemental Experimental Procedures and Figures S1–S4

Document S2. Article plus Supplemental Information
