# Supplementary material for: Fission Yeast Apc15 Stabilizes MCC-Cdc20-APC/C Complexes, Ensuring Efficient Cdc20 Ubiquitination and Checkpoint Arrest
Source: Curr Biol. 2017 Apr 24;27(8):1221–8. doi: 10.1016/j.cub.2017.03.013 (PMC5405113; doi:10.1016/j.cub.2017.03.013)
Supplement: Document S1. Supplemental Experimental Procedures and Figures S1–S4 [file mmc1.pdf]

Current Biology, Volume *27*

Supplemental Information

Fission Yeast Apc15 Stabilizes MCC-Cdc20-APC/C Complexes, Ensuring Efficient Cdc20 Ubiquitination and Checkpoint Arrest

Karen M. May, Flora Paldi, and Kevin G. Hardwick

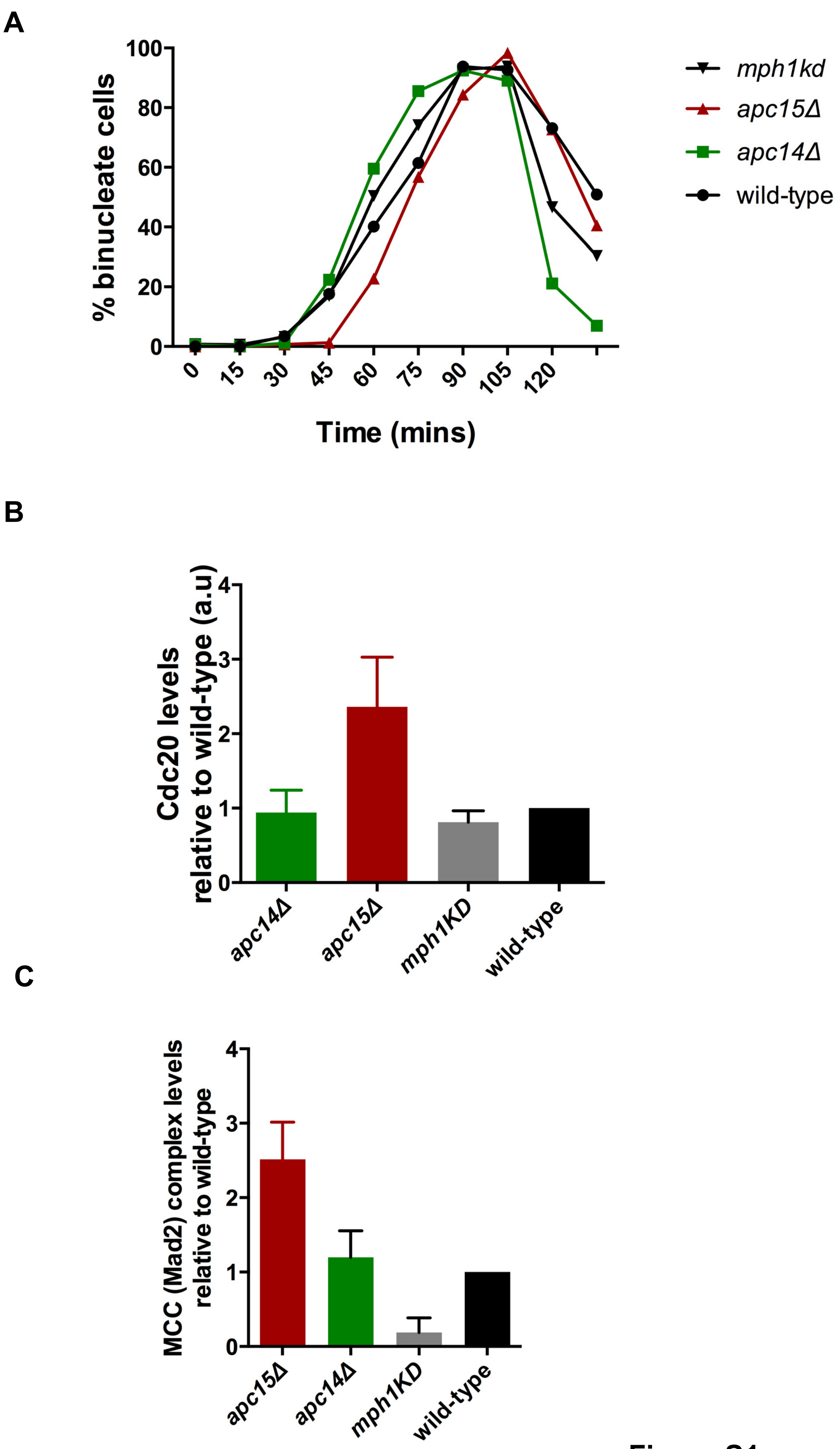
A
% binucleate cells
100
80
60
40
20
0
0
15
30
45
60
75
90
105
120
Time (mins)
mph1kd
apc15Δ
apc14Δ
wild-type
B
Cdc20 levels
relative to wild-type (a.u)
4
3
2
1
0
apc14Δ
apc15Δ
mph1KD
wild-type
C
MCC (Mad2) complex levels
relative to wild-type
4
3
2
1
0
apc15Δ
apc14Δ
mph1KD
wild-type

Figure S1

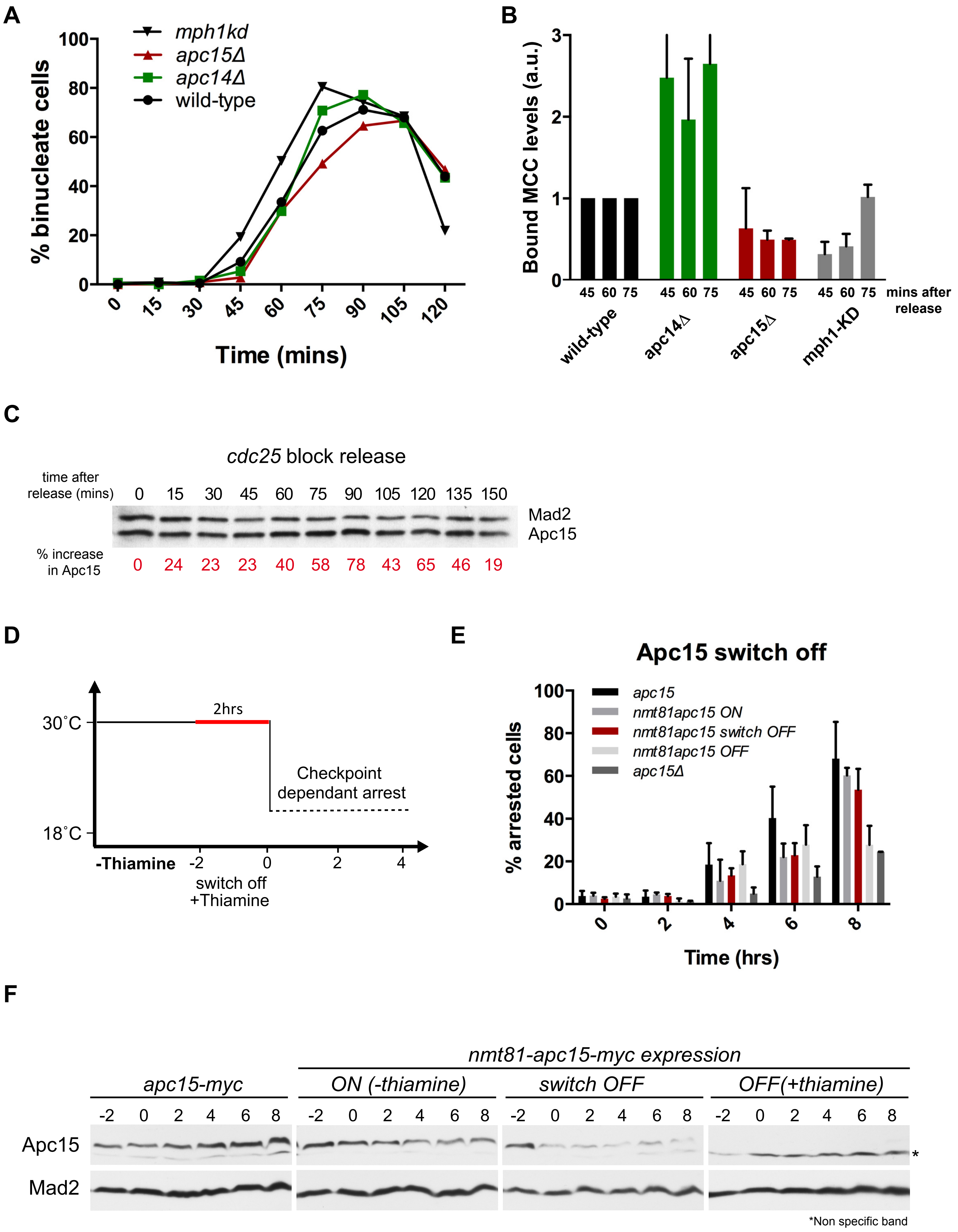
A
% binucleate cells
Time (mins)
mph1kd
apc15Δ
apc14Δ
wild-type
0 20 40 60 80 100
0 15 30 45 60 75 90 105 120
B
Bound MCC levels (a.u.)
0 1 2 3
45 60 75
mins after release
wild-type
apc14Δ
apc15Δ
mph1-KD
C
cdc25 block release
time after release (mins)
0 15 30 45 60 75 90 105 120 135 150
Mad2
Apc15
% increase in Apc15
0 24 23 23 40 58 78 43 65 46 19
D
30˚C
18˚C
2hrs
Checkpoint dependant arrest
-Thiamine
-2 0 2 4
switch off +Thiamine
E
Apc15 switch off
% arrested cells
Time (hrs)
apc15
nmt81apc15 ON
nmt81apc15 switch OFF
nmt81apc15 OFF
apc15Δ
0 20 40 60 80 100
0 2 4 6 8
F
nmt81-apc15-myc expression
apc15-myc
ON (-thiamine)
switch OFF
OFF(+thiamine)
-2 0 2 4 6 8
Apc15
Mad2
*
*Non specific band

Figure S2

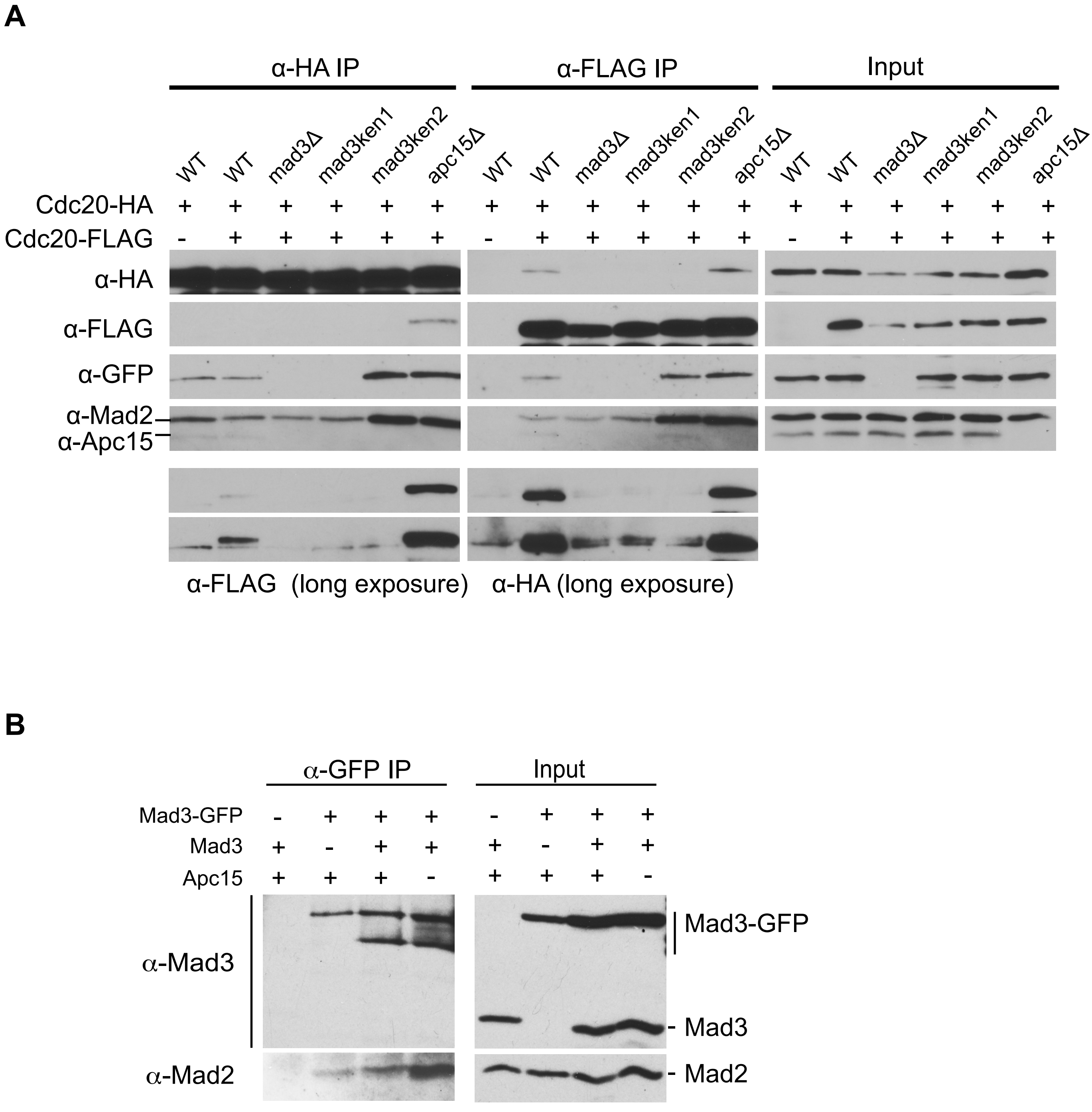
A
α-HA IP
α-FLAG IP
Input
WT
WT
mad3Δ
mad3ken1
mad3ken2
apc15Δ
Cdc20-HA
Cdc20-FLAG
α-HA
α-FLAG
α-GFP
α-Mad2
α-Apc15
α-FLAG (long exposure)
α-HA (long exposure)
B
α-GFP IP
Input
Mad3-GFP
Mad3
Apc15
α-Mad3
α-Mad2
Mad3-GFP
Mad3
Mad2

Figure S3

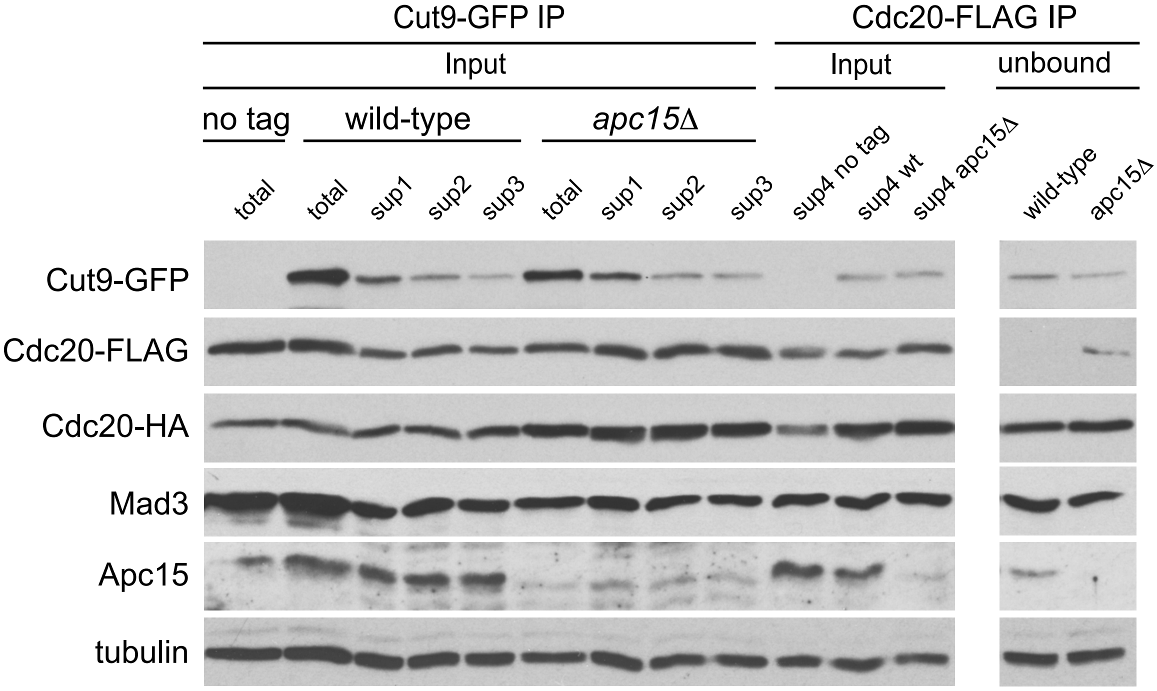
Cut9-GFP IP
Cdc20-FLAG IP
Input
Input
unbound
no tag
wild-type
apc15Δ
total
total
sup1
sup2
sup3
total
sup1
sup2
sup3
sup4 no tag
sup4 wt
sup4 apc15Δ
wild-type
apc15Δ
Cut9-GFP
Cdc20-FLAG
Cdc20-HA
Mad3
Apc15
tubulin

Figure S4

Figure S1, related to Figure 1C

(A) *cdc25* strains containing *wild-type*, *apc14Δ*, *apc15Δ* and *mph1-kd* were synchronised in G2 then released in mitosis. Progression through mitosis was monitored by scoring the number of binucleate cells. This shows that the majority of cells in Figure 1C had entered anaphase by 90mins.

(B) Cdc20 levels are increased in *apc15*Δ. The levels of Cdc20 in lysates from Figure 1C were quantitated and normalised for loading. The plot shows the amount of Cdc20, relative to the level in wild-type, at the time points when Cdc20 levels peaked in each mutant. Plotted as mean with standard deviation (n = 3 experiments).

(C) MCC levels are increased in *apc15*Δ. The levels of the MCC subunit Mad2 in immunoprecipitations from Figure 1C were quantitated and normalised against immunoprecipitated Cdc20 levels. The plot shows the amount of Mad2 relative to the level in wild-type at the time points when Cdc20 levels peaked in each mutant. Plotted as mean with standard deviation (n = 3 experiments).

Figure S2, related to Figure 2

(A) *cdc25* strains along with those containing *apc14Δ*, *apc15Δ* or *mph1-kd* were synchronised in G2 then released into mitosis. Progression through mitosis was monitored by scoring the number of binucleate cells. This data shows that the majority of cells in Figure 2A had entered anaphase by 75mins, and had completed mitosis by 120mins.

(B) Quantitation of MCC levels bound to APC/C. The levels plotted for the 3 strains are the combined levels of bound Mad3 and Mad2, relative to wild-type levels (set at 1.0), at the three time points indicated after *cdc25* block/release. This experiment was repeated twice and data is plotted as the mean +/- SD.

(C) Apc15 levels increase in mitosis. Cultures were synchronised at G2/M by *cdc25* block and release, cell samples taken at 15 minute intervals and immunoblots analysed for levels of Apc15 and Mad2 as a loading control. This experiment was repeated 4 times and a representative example is shown here. The fold increase of Apc15 levels is shown for each time point and the data is plotted as the mean +/- SD. Previous work has shown that whilst the abundance of several APC/C sub-units remain constant through the cell cycle (e.g. Nuc2 and Cut9 [S1]) others (eg. Cut23 [S2]) also increase in mitosis.

(D) Apc15 'switch OFF' experimental scheme. *nda3-KM11* cells with *apc15* under the control of the inducible nmt81 promoter were grown in minimal media with (Apc15 OFF) or without (Apc15 ON) thiamine. To 'switch off' Apc15 expression the Apc15 ON culture was split into 2 flasks and thiamine added to one of the flasks (Apc15 Switch OFF), then all were incubated for 2hrs at 30°C before shifting their temperature to 17.5^{o}C to induce mitotic arrest. Samples were taken every 2hrs and processed for protein extraction and microscopy.

(E) Mitotic expression of Apc15 is not required for checkpoint arrest. The *nda3* mutant Apc15 ON, Apc15 OFF and Apc15 'switch off' cultures (as described in S3C), along with *apc15*$^{+}$ and *apc15Δ* controls, were shifted to 17.5^{o}C to induce mitotic arrest. Their ability to arrest was determined by cdc13-GFP localisation (this cyclin B accumulates in nuclei and at spindle poles in arrested cells). *apc15Δ* and Apc15 OFF did not arrest, but the Apc15 'switch' OFF strain was able to arrest as well as Apc15 ON and *apc15+* expressed from the endogenous promoter.

(F) Protein extracts from the cells in Figure S3D were prepared and Apc15 levels analysed by immunoblotting (anti-Apc15). No Apc15 was detected in Apc15 OFF (* indicates a non-specific band recognised by the anti-Apc15 antibody). Importantly, the Apc15 levels in Apc15 ON were comparable to levels expressed from the endogenous promoter and the Apc15 levels had decreased 2 hrs after expression was repressed (Switch OFF) and thus before the checkpoint was activated using the *nda3* mutant.

Figure S3, related to Figure 3B

(A) Cdc20-FLAG and Cdc20-HA co-immunoprecipitate (related to Figure 4A). Cells containing both Cdc20 forms were synchronised in mitosis (60 mins post *cdc25* block & release), lysates prepared and Cdc20-FLAG or Cdc20-HA was immunoprecipitated. The immunoprecipitates were then immunoblotted and analysed for associated Cdc20-HA (or Cdc20-FLAG), Mad3-GFP, Mad2 and Apc15. The second Cdc20 form is associated

with each immunoprecipitated Cdc20, and *mad3* mutants abolish this interaction. Importantly, the interaction with between two Cdc20 molecules is dependent on KEN2, as well as KEN1. It is not dependent on Apc15. This experiment was repeated 3 times.

(B) Mad3 and Mad3-GFP do not co-immunoprecipitate. Cells expressing both Mad3 forms were synchronised in mitosis (60 mins post *cdc25* block & release), lysates prepared and Mad3-GFP was immunoprecipitated. No untagged Mad3 protein was detectable in these IPs. This argues against the presence of MCC dimers.

Figure S4, related to Figure 4A

(A) Mitotic lysates from Figure 4A. These were immunoblotted for the indicated proteins, with tubulin being used as a loading control.

Supplemental Experimental Procedures

Deletion of *apc14* and *apc15*

To replace the entire open reading frame with NatR, KanR or HphR, the resistance cassettes were amplified from pFA6 vectors by PCR using compatible long primers [S3] with 5' sequences homologous to the 5' and 3' UTRs of *apc14* or *apc15*. Resulting PCR products were transformed [S4] into RA366 and the gene deletions confirmed by PCR.

Mitotic arrests

nda3-KM311(cs) cells were grown overnight in YES media at 30°C to mid-log phase then shifted to the restrictive temperature of 17.5^{o}C. Cells were fixed in 100% ice cold methanol at each time point. For microscopy, cells were mounted with 20 µg/ml DAPI (Sigma) and visualized using an Intelligent Imaging Innovations Marianas microscope (Zeiss Axiovert 200M, using a 100x 1.3NA objective lens), CoolSnap CCD, and Slidebook software (Intelligent Imaging Innovations, Inc., Boulder, CO).

Mph1 was overexpressed [S5] from the nmt41 promotor at the endogenous locus. To induce Mph1 expression cells were precultured in minimal medium with supplements (PMGS) and 15µM thiamine, cells were washed 3 times in H_20 to remove thiamine then grown in PMGS at 30°C for 18 hours before analysis. Mitotic arrest was determined by immunostaining microtubules with TAT1 antibody (kindly provided by Keith Gull, Oxford, UK). Mad2 was overexpressed from the multicopy pRep1-Mad2 in the same way [S6].

To synchronise cells in mitosis temperature sensitive (ts) *cdc25-22* cells were grown overnight in YES medium at 25°C to mid-log phase and then shifted to 36°C for 3.5 hrs to arrest cells in G2. To release into mitosis, cultures were rapidly cooled down to 25°C in iced-water and then incubated at 25°C for the experimental time course. Progression through mitosis was monitored by DAPI staining and counting binucleate cells.

Silencing Assay

The silencing assay was carried out as in [S7]. Briefly, cells were arrested in metaphase using *nda3-KM311* grown at 17.5°C for 5.5hrs 5µM 1NM-PP1 was added and the degradation of Cdc13-GFP analysed by microscopy of live cells every 15mins. The number of cells degrading Cdc13-GFP was determined as a percentage of the number of metaphase arrested cells before 1NM-PP1 addition.

Immunoprecipitations

*MCC (*Cdc20^{Slp1}-FLAG) *immunoprecipitation*

Cells expressing Cdc20^{Slp1}-FLAG [S8] were pre-synchronised in G2 by *cdc25-22* arrest then release into mitosis. Harvested cells were pelleted at 4°C and snap frozen on dry ice. Proteins were extracted in lysis buffer (50 mM HEPES pH 7.5, 75 mM KCl, 1 mM $MgCl_2$, 1 mM EGTA, 0.1% TritonX-100, 1 mM sodiumvanadate, 0.1 µM microcystin, 10 µg/ml CLAAPE (chymostatin, leupeptin, aprotinin antipain pepstatin, E64) 'mini complete EDTA-free protease inhibitor' tablet (Roche) and 1 mM pefabloc). Cells were resuspended in lysis buffer and bead-beaten twice for 20 seconds. Extracts were incubated for 20 min with anti-FLAG (M2, Sigma) antibodies that had been pre-coupled to Dynabeads (Invitrogen). The immunoprecipitated complexes were washed three times with lysis buffer, twice in PBS and then analysed by immunoblotting with sheep anti-GFP antibody and sheep anti-Mad2 antibody.

Anaphase promoting complex interaction

Cells expressing TAP-tagged Lid1 (Apc4) (original strain kindly provided by Kathy Gould, Vanderbilt, USA) immunoprecipitated as above, but were incubated for 30 min with IgG-coupled Dynabeads (Invitrogen), which bind to Apc4-TAP.

Blot quantitation

Samples from all strains were collected on one day and then analysed the next day in parallel. Time course samples were processed at the same time and the full set of IPs were run on gels and transferred to membrane in parallel (whole cell lysates were typically run the following day). eg. Membrane organisation for Fig 1: wild-type and *apc14*Δ IPs were on one membrane, *mph1-KD* and *apc15*Δ IPs on another. The input lysates were run and transferred to membranes as for the IPs. Note, for Fig1 the same membrane was first probed with anti-FLAG (Cdc20^{Slp1}) and anti-Mad2, then stripped and re-probed with anti-GFP (Mad3), so one pipetted IP sample was being analysed for all 3 proteins. For Fig2, anti-GFP antibody recognised lid1-TAP, Mad2-GFP and Mad3-GFP. For each antibody all of the membranes were exposed at the same time to a single piece of film for each exposure.

Immunoprecipitates from such time courses were analysed by ImageJ and corrected for loading (and normalized for the level of $Cdc20^{Slp1}$ in the IP).

For analysis of $Cdc20^{Slp1}$-ubiquitin
Cells were synchronised in mitosis as above and harvested 60 min after release from the G2/M block. Lysates were prepared as above with addition of 10mM MG132, 40mM PR-619 200mM 1,10-phenanthroline, 500mM iodoacetamide and 400mN NEM to the lysis buffer.

Analysis of $Cdc20^{Slp1}$- $Cdc20^{Slp1}$complexes
A second copy of Cdc20 was inserted in the genome by integrating c*dc20^{Slp1}-FLAG* with 5' and 3' regulatory regions into the *ura4* locus. An 800bp *cdc20+* promoter sequence and the *cdc20-FLAG-hyg+* open reading frame (with 3'UTR sequences) was amplified by PCR and cloned to the Stu1 site of pBluescript-*ura4+* by Gibson assembly reaction (Gibson *et al*., 2009). Endogenous *cdc20^{Slp1}* was HA tagged. Cells were synchronised in mitosis and lysates prepared as above, then extracts were incubated for 20 min with anti-FLAG (M2, Sigma) or anti-HA (12CA5) antibodies that had been pre-coupled to Dynabeads (Invitrogen).

APC depletions
nda3-KM311 apc1-GFP cells were grown at 30°C in concentrated rich medium to high densities then arrested in mitosis by rapid cooling on iced-water and shifting cultures to 18°C for 8 hours. Cells were harvested by centrifugation at 3,500g for 8 minutes at 4°C. Pelleted cells were frozen into pea-sized drops using liquid nitrogen and stored at -80°C. Approximately 15 g of cell mass was disrupted using a mixing mill (MM400; Retsch, Germany) with grinding balls under cryogenic conditions (5 cycles of 3 minutes at 30 Hz).

Lysates were reconstituted with 2.5g of yeast powder and 5ml lysis buffer (50 mM HEPES pH 7.5, 75 mM KCl, 1 mM $MgCl_2$, 1 mM EGTA, 1 mM sodium vanadate, 0.1 µM microcystin, 10 µg/ml CLAAPE (chymostatin, leupeptin, aprotinin antipain pepstatin, E64), a 'mini complete EDTA-free protease inhibitor' tablet (Roche) and 1 mM Pefabloc). Triton X100 (0.1% final) was added after lysates were resuspended. Following further lysis, by sonication, cell debris was pelleted at 14,000g for 15 min at 4°C and the supernatant was filtered through a 1.6 µm syringe filter. Cleared lysates were incubated with precoupled anti-GFP antibodies, that had been pre-coupled to Dynabeads (Invitrogen), for 30 min. The resulting supernatant was incubated with fresh GFP Dynabeads for 30min and Dynabeads with bound proteins were washed three times with lysis buffer before eluting proteins in 1x SDS sample buffer. This process was repeated 3 times, then the 4th supernatent was incubated with pre-coupled anti-Apc15 Dynabeads for 30 min at 4°C

$Apc6^{Cut9}$-GFP depletions were carried out as above except, *cdc25-22 cut9-GFP* cells were grown at 25°C, synchronised at G2/M, then cells were harvested 60min after release into mitosis. Lysates were prepared as for MCC immunoprecipitation by bead beating 0.2g cell pellets twice for 30 sec in lysis buffer with 0.1% TritonX-100. Lysates were incubated for 15 min with anti-GFP antibodies that had been pre-coupled to Dynabeads (Invitrogen) and then 20min with anti-FLAG (M2, Sigma) Dynabeads.

Apc15 Switch OFF
The *apc15* promotor was replaced by the nmt81 promotor. Cells were grown in PMGS –thiamine at 30°C for 24 hours to ensure full expression of Apc15, then 15uM thiamine was added to repress transcription of Apc15. The cells were incubated for a further 2 hrs to ensure complete repression before inducing mitotic arrest using the cold sensitive *nda3-KM311* mutation. Samples were taken every 2 hrs and snap frozen on dry ice for protein extraction and immunoblot analysis of Apc15 levels, or fixed in ice cold methanol for microscopy. Ability to arrest in mitosis was determined by Cdc13-GFP localisation.

Apc15 antibodies
Polyclonal anti-Apc15 antibodies were generated in sheep using 6xHis-Apc15 as antigen. These sera were affinity purified as described previously [S9] using Apc15-MBP coupled to Affigel 10 (Bio-Rad).

List of fission yeast strains used in this study

Figure 1

JZ108	*nda3-KM311 cdc13-GFP::leu2+ leu1-32 ura4D18*	This lab
KM905	*nda3-KM311 cdc13-GFP::leu2 apc14Δ::kanR leu1-32 ura4D18*	This lab
KM916	*nda3-KM311 cdc13-GFP::leu2 apc15Δ::kanR leu1-32 ura4D18*	This lab
VV1369	*nda3-KM311 cdc13-GFP::leu2 mad2Δ::kanR leu1-32 ura4D18*	This lab
KM936	*nda3-KM311 cdc13-GFP::leu2 mad2Δ::kanR apc15Δ::kanR leu1-32 ura4D18*	This lab
KM910	*nmt41Mph1::NAT leu1-32 ura4D18*	
KM903	*nmt41Mph1::NAT apc14Δ::kanR leu1-32 ura4D18*	This lab
KM910	*nmt41Mph1::NAT apc15Δ::kanR leu1-32 ura4D18*	This lab
KP114	*leu1-32 ura4D18 ade6-216*	Allshire
KM888	*apc15Δ::kanR leu1-32 ura4D18/ pRep1-Mad2*	This lab
OS18	*cdc25-22 slp1-FLAG::hph mad3-GFP::*$his3^{+}$ *leu1-32 ura4D18*	This lab
KM1218	*cdc25-22 slp1-FLAG::hph mad3-GFP::*$his3^{+}$ *mph1-kd::*$leu1^{+}$ *leu1-32 ura4D18*	This lab
KM1220	*cdc25-22 slp1-FLAG::hph mad3-GFP::*$his3^{+}$ *apc15Δ::NAT leu1-32 ura4D18*	This lab
KM1273	*cdc25-22 slp1-FLAG::hph mad3-GFP::*$his3^{+}$ *apc14Δ::NAT leu1-32 ura4D18*	This lab

Figure 2

MS304	*cdc25-22 lid1-TAP::*Kan^{r} *mad3-GFP::*$his3^{+}$ *leu1-32 ura4D18*	This lab
YJZ917	*cdc25-22 lid1-TAP::*Kan^{r} *mad3-GFP::*$his3^{+}$ *mph1-kd::*$leu1^{+}$ *leu1-32 ura4D18*	This lab
KM896	*cdc25-22 lid1-TAP::*Kan^{r} *mad3-GFP::*$his3^{+}$ *apc15Δ::NAT leu1-32 ura4D18*	This lab
KM890	*cdc25-22 lid1-TAP::*Kan^{r} *mad3-GFP::*$his3^{+}$ *apc14Δ::NAT leu1-32 ura4D18*	This lab
VV1381	*ark1-as3::hyg cdc13-gfp::leu nda3-KM11 leu1-32 ura4D18*	This lab
VV1403	*ark1-as3::hyg cdc13-gfp::leu nda3-KM11 bub3::ura4+ leu1-32 ura4D18*	This lab
KM870	*ark1-as3::hyg cdc13-gfp::leu nda3-KM11 apc14Δ::kanR leu1-32 ura4D18*	This lab
VV54	*nda3-KM311 leu1-32 ura4D18*	
KM1160	*nda3-KM311* $apc1^{cut4}$-GFP::kanR *leu1-32 ura4D18*	This lab

Figure 3

KM1146	*cdc25-22 slp1-FLAG::hph mad3-GFP::*$his3^{+}$ *leu1-32 ura4D18*	This lab
KM1319	*mts3-1 slp1-FLAG::hygR leu1-32 ura4D18*	This lab
KM1320	*mts3-1 slp1-FLAG::hygR apc15Δ::NAT leu1-32 ura4D18*	This lab
KM1321	*mts3-1 slp1-FLAG::hygR apc14Δ::NAT leu1-32 ura4D18*	This lab
KM1336	*mts3-1 slp1-FLAG::hygR apc15Δ::NAT mad3-KEN2-GFP::his3 leu1-32 ura4D18*	This lab
KM1344	*mts3-1 slp1-FLAG::hygR apc15Δ::NAT mad3Δ::ura4 leu1-32 ura4D18*	This lab
KM1358	*mts3-1 slp1-FLAG::hygR mad3Δ::ura4 leu1-32 ura4D18*	This lab
KM1364	*mts3-1 slp1-FLAG::hygR apc15Δ::NAT mad3-KEN1-GFP::his3 leu1-32 ura4D18*	This lab
FP3	*cdc25-22 slp1-HA::KanR ura4::slp1-FLAG::hygR mad3-GFP::*$his3^{+}$ *leu1-32 ura4D18*	This lab
KM1313	*cdc25-22 slp1-HA::KanR ura4::slp1-FLAG::hygR mad3Δ::ura4 leu1-32 ura4D18*	This lab
KM1314	*cdc25-22 slp1-HA::KanR ura4::slp1-FLAG::hygR mad3-KEN2-GFP::his3 leu1-32 ura4D18*	This lab
KM1315	*cdc25-22 slp1-HA::KanR ura4::slp1-FLAG::hygR apc15Δ::NAT mad3-GFP::*$his3^{+}$ *leu1-32 ura4D18*	This lab
KM1316	*cdc25-22 slp1-HA::KanR ura4::slp1-FLAG::hygR mad3-KEN1-GFP::his3 leu1-32 ura4D18*	This lab

Figure 4

KM1465	*cdc25-22 slp1-HA::KanR ura4::slp1-FLAG::hygR* $cut9^{apc6}$*-GFP::kanR leu1-32 ura4D18*	This lab
KM1466	*cdc25-22 slp1-HA::KanR ura4::slp1-FLAG::hygR* $cut9^{apc6}$*-GFP::kanR apc15Δ::NAT leu1-32 ura4D18*	This lab

Figure S2

KM1179	*nda3-KM311 cdc13-GFP::leu1 nat:nmt81-apc15-myc::kanR*	This lab

Figure S3

KM1470	*cdc25-22* $mad3^{+}$ *ura4D18*	This lab
KM1471	*cdc25-22 mad3Δ::ura4 leu1:: mad3-GFP ura4D18*	This lab

KM1472	*cdc25-22 mad3⁺ leu1:: mad3-GFP ura4D18*	This lab
KM1473	*cdc25-22 mad3⁺ leu1:: mad3-GFP apc15Δ::NAT ura4D18*	This lab

Supplemental References:

S1. Yamada, H., Kumada, K., and Yanagida, M. (1997). Distinct subunit functions and cell cycle regulated phosphorylation of 20S APC/cyclosome required for anaphase in fission yeast. J Cell Sci *110 (Pt 15)*, 1793-1804.

S2. Yamashita, Y.M., Nakaseko, Y., Kumada, K., Nakagawa, T., and Yanagida, M. (1999). Fission yeast APC/cyclosome subunits, Cut20/Apc4 and Cut23/Apc8, in regulating metaphase-anaphase progression and cellular stress responses. Genes Cells *4*, 445-463.

S3. Bahler, J., Wu, J.Q., Longtine, M.S., Shah, N.G., McKenzie, A., 3rd, Steever, A.B., Wach, A., Philippsen, P., and Pringle, J.R. (1998). Heterologous modules for efficient and versatile PCR-based gene targeting in Schizosaccharomyces pombe. Yeast *14*, 943-951.

S4. Ito, H., Fukuda, Y., Murata, K., and Kimura, A. (1983). Transformation of Intact Yeast Cells Treated with Alkali Cations. J Bacteriol *153*, 163-168.

S5. He, X., Jones, M.H., Winey, M., and Sazer, S. (1998). Mph1, a member of the Mps1-like family of dual specificity protein kinases, is required for the spindle checkpoint in S. pombe. J Cell Sci *111 (Pt 12)*, 1635-1647.

S6. He, X.W., Patterson, T.E., and Sazer, S. (1997). The Schizosaccharomyces pombe spindle checkpoint protein mad2p blocks anaphase and genetically interacts with the anaphase-promoting complex. Proceedings of the National Academy of Sciences of the United States of America *94*, 7965-7970.

S7. Vanoosthuyse, V., and Hardwick, K.G. (2009). A novel protein phosphatase 1-dependent spindle checkpoint silencing mechanism. Curr Biol *19*, 1176-1181.

S8. Zich, J., May, K., Paraskevopoulos, K., Sen, O., Syred, H.M., van der Sar, S., Patel, H., Moresco, J.J., Sarkeshik, A., Yates, J.R., 3rd, et al. (2016). $Mps1^{Mph1}$ Kinase Phosphorylates Mad3 to Inhibit $Cdc20^{Slp1}$-APC/C and Maintain Spindle Checkpoint Arrests. PLoS Genet *12*, e1005834.

S9. Hardwick, K.G., and Murray, A.W. (1995). Mad1p, a phosphoprotein component of the spindle assembly checkpoint in budding yeast. J. Cell Biol. *131*, 709-720.
